# Identifying microbial proteins and changes in proteome in spontaneously fermented pulse protein isolates

**DOI:** 10.1016/j.fochms.2025.100254

**Published:** 2025-03-14

**Authors:** Prem Prakash Das, Caishuang Xu, Yuping Lu, Enyu Liu, Zahra Jafarian, Takuji Tanaka, Darren Korber, Michael Nickerson, Nandhakishore Rajagopalan

**Affiliations:** a National Research Council Canada, 110 Gymnasium Pl, Saskatoon, SK S7N 0W9, Canada; b University of Saskatchewan, Department of Food and Bioproduct Sciences, 51 Campus Drive Saskatoon, SK S7N 5A8, Canada; c University of Saskatchewan, Department of Chemical and Biological Engineering, 57 Campus Drive Saskatoon SK, S7N 5A9, Canada

**Keywords:** Chickpea, Faba bean, Green lentil, Proteome, Foodomics, Solid state fermentation

## Abstract

Pulses are a sustainable source of plant-based proteins, but they often fall short in terms of sensory attributes and functionality. Fermentation has been investigated as a natural food processing method to address these limitations. Spontaneous fermentation, where native microflora grow without the addition of specific microbes, has been traditionally used in food processing by various cultures around the world. However, there is a knowledge gap regarding the changes that occur in protein composition during spontaneous fermentation. This study used capillary gel electrophoresis and liquid chromatography coupled to tandem mass spectrometry to examine the changes in protein size distribution, identify microbial proteins and understand proteome-level changes that occurred during the spontaneous fermentation of three protein isolate substrates: chickpea, faba bean, and lentil. The findings revealed that proteins from a variety of bacterial and fungal species were identified in all substrates, and the number and quantity of these microbial proteins increased during spontaneous fermentation. This rise in microbial protein content was associated with the hydrolysis of proteins from the pulse substrates, which could potentially alter the functionality of the protein ingredient.

## Highlights

• First proteomics study of spontaneous fermentation of chickpea, lentil and faba bean.
• Identification of diverse proteins produced by indigenous microflora.
• Changes in the proteome of substrate and indigenous microflora during fermentation.
• Identified variations between substrates in their indigenous microbial protein content.

## Graphical abstract

**Figure:**
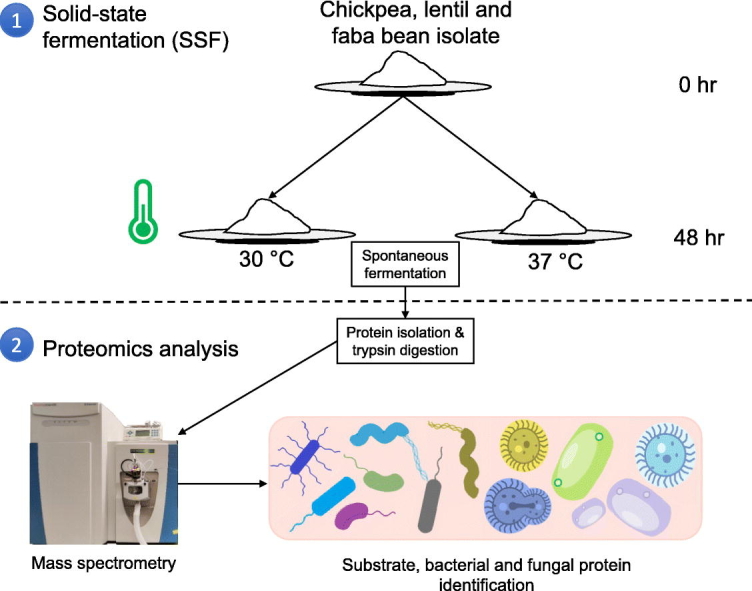

## 1 Introduction

Pulses are a sustainable, inexpensive, and nutritionally complete source of plant-based proteins. Pulses are the dry seeds of legume crops, such as chickpeas (*Cicer arietinum* L.), peas (*Pisum sativum* L.), faba beans (*Vicia faba* L.), and lentils (*Lens culinaris*), as defined by the Food and Agriculture Organization of the United Nations. These crops are notably rich in protein content, fiber, minerals, and vitamins (Bouchenak & Lamri-Senhadji, 2013; Clemente & Olias, 2017). Moreover, numerous studies have underscored the manifold health benefits associated with regular pulse protein consumption, such as a reduced risk of heart disorders, cancer, diabetes, hypertension, and improvement of gut health (Hall et al., 2017; Samtiya et al., 2021; Singh et al., 2017). Despite their benefits, the use of pulse proteins is limited by factors such as poor techno-functional properties, presence of anti-nutritional compounds, allergenic epitopes, and an unfavorable sensory profile. To improve aspects like flavor and texture and reduce anti-nutritional components, fermentation technology has been explored as an effective natural food processing technique (Ding et al., 2023; Khorsandi, Shi, et al., 2023; Khorsandi, Stone, et al., 2023). Fermentation is an age-old food processing technique that involves modification of food quality through the action of specific microorganisms, such as yeast, filamentous fungi, and bacteria. Fermentation can be conducted as submerged fermentation, which involves a liquid culture with abundant free water and nutrients, or as solid-state fermentation (SSF), where water availability is scarce (Pandey, 2003; D. Shi et al., 2024). Submerged fermentation is extensively utilized in the production of various food products such as vinegar, alcoholic beverages like beer and wine, and dairy products like yogurt. In contrast, SSF is used in making food products like tempeh, sufu, and natto (Canoy et al., 2024). Additionally, fermentation can be carried out either as inoculated fermentation, where specific microorganisms such as (*Aspergillus* sp., Lactic acid bacteria, etc.) are intentionally introduced, or as spontaneous fermentation, which relies on growth of naturally occurring endogenous microbes (Canonico et al., 2022; Tamang et al., 2016). The spontaneous fermentation process relies on the presence of native microorganisms or spores in the substrate (seed, flour, isolate, etc.) and favourable environmental conditions to initiate and sustain fermentation. Spontaneous fermentation has been used intentionally for the processing of many traditional foods such as idli (Agaliya & Jeevaratnam, 2013), adai (Ray et al., 2016), kimchi (Lee et al., 2020), sauerkraut (Yang et al., 2020), and rice wine (Dung et al., 2007), by various cultures around the world. The growth of microorganisms on substrates and the production of hydrolytic enzymes and other compounds causes changes in metabolic, biochemical, and biophysical properties, thereby enhancing the techno-functional, sensory, and nutritional characteristics of food products (Adebo et al., 2022; El Youssef et al., 2020; Kumitch et al., 2020).

Some recent studies have tried to identify the microbial proteins that are produced during fermentation of substrates inoculated with known microbial strains, as well as characterize the changes that the substrate proteome undergoes during fermentation (Das et al., 2023; Tahmasian et al., 2023). However, the composition of proteins produced by native microbial species present in the substrate, as well as the modifications to the substrate and microbial proteomes after spontaneous fermentation remains unexplored. Such information regarding the composition of microbial populations, as well as the proteins that they add to the processed substrate are vital for ensuring quality and safety of novel food products that are being developed through spontaneous fermentation processing. The main objectives of the current work are to identify the proteins originating from native microbial populations present in the substrate before and after spontaneous fermentation, then to characterize the changes in the size profiles and composition of the substrate and microbial proteins and finally, to understand the differences between the proteomes of three common pulse protein substrates that are processed by spontaneous fermentation. The changes in protein size distribution due to spontaneous fermentation of pulse protein isolate substrates was studied using capillary gel electrophoresis (CGE) and the identity of proteins and the proteome-wide changes were characterized by employing the liquid chromatography-tandem mass spectrometry (LC-MS/MS) shotgun proteomics approach. The map of changes to protein composition in spontaneously fermented matrix generated in the current study will complement other efforts to identify native microbial populations and support the development of safe and sustainable processing technologies for the production of functional food ingredients.

## 2 Materials and methods

### 2.1 Materials

Triethylammonium bicarbonate (TEAB) (catalog number T7408) and sodium dodecyl sulfate (SDS) (catalog number 71736) were purchased from Sigma-aldrich® (St. Louis, MO, USA). Protein quantification was performed using the RC DC™ Protein Assay (catalog number 5000121) (Bio-Rad, Mississauga, ON, Canada). Protein digestion was carried out using S-Trap™ mini columns (ProtiFi, NY, USA). Peptide quantification was done using the Pierce™ Quantitative Colorimetric Peptide Assay (catalog number 23275) (Thermo Scientific™, Mississauga, ON, Canada). Protein isolates of chickpea, green lentil, and faba bean were obtained from commercial sources. The protein content of the chickpea, lentil and faba bean isolates were 73 %, 82 % and 88 %, respectively.

### 2.2 Spontaneous fermentation of substrates

Protein isolates from chickpea, faba bean and lentils (∼60 g) were used for solid-state fermentation (see Table 1 for sample information). The solid-state fermentation conditions used for the modification of these substrates have been described in detail recently (Stone et al., 2024). Briefly, the protein isolates were mixed with sterilized water to an initial moisture content of 50 % (*w*/w). The dough substrate was spread evenly on an aluminium baking tray (length x width x height of 13 x 9 x 2 inches). The pans were covered with an aluminium foil and allowed to ferment spontaneously at 30 °C or 37 °C for 48 h in an Isotemp incubator, Model 650D (Thermo Fisher Scientific™, Mississauga, ON, Canada). The moisture content throughout the fermentation period was maintained close to the starting point by adding sterilized water as required to compensate for weight loss due to evaporation. After 48 h of fermentation time the doughs were dried in a heated air circulated oven set at 63 °C for 24 h and the dried material was powdered using a bench top mill (Cuisinart DBM-8C coffee burr mill, Woodbridge, ON, Canada). Samples collected at 0 h and 48 h time-points of uninoculated spontaneous fermentation at 30 °C and 37 °C from the Stone et al., 2024 work were used for the detailed proteomics analysis in the current study (Stone et al., 2024). Each protein isolate substrate fermentation was independently repeated twice. For subsequent proteomics analysis, each sample collected from independent fermentation replicates were each analyzed further as two technical replicates.

**Table 1:** List of controls and spontaneously fermented samples used for the study.

Pulse type	Sample name	Time-point	Fermentation Temperature	Replicate
Chickpea	VSC1	0 h-ctrl (control)	N/A	1
Chickpea	VSC2	0 h-ctrl (control)	N/A	2
Chickpea	VSC3	48 h-30 °C	30 °C	1
Chickpea	VSC4	48 h-30 °C	30 °C	2
Chickpea	VSC5	48 h-37 °C	37 °C	1
Chickpea	VSC6	48 h-37 °C	37 °C	2
Green lentil	VSL1	0 h-ctrl (control)	N/A	1
Green lentil	VSL2	0 h-ctrl (control)	N/A	2
Green lentil	VSL3	48 h-30 °C	30 °C	1
Green lentil	VSL4	48 h-30 °C	30 °C	2
Green lentil	VSL5	48 h-37 °C	37 °C	1
Green lentil	VSL6	48 h-37 °C	37 °C	2
Faba bean	VSF1	0 h-ctrl (control)	N/A	1
Faba bean	VSF2	0 h-ctrl (control)	N/A	2
Faba bean	VSF3	48 h-30 °C	30 °C	1
Faba bean	VSF4	48 h-30 °C	30 °C	2
Faba bean	VSF5	48 h-37 °C	37 °C	1
Faba bean	VSF6	48 h-37 °C	37 °C	2

*Note*: N/A, not applicable.

### 2.3 Capillary gel electrophoresis (CGE)

Proteins from the control and spontaneously fermented samples from all the substrates were analyzed based on their molecular weight by CGE. Total protein was extracted for CGE from control and fermented protein isolate samples of each legume using a buffer containing 100 mM tris(hydroxymethyl)aminomethane (Tris) pH 9.0 and sodium dodecyl sulfate (SDS) at 1:10 ratio (w:v, protein isolates: buffer) and incubated for 30 min at room temperature while shaking in mixer. After centrifugation at 15,000 ×*g* for 10 min, the soluble protein supernatants were transferred to clean tubes. A P/ACE™ MDQ plus electrophoresis system (Sciex, Framingham, U.S.A.) coupled with a UV detector was utilized to perform the CGE experiment. Data acquisition and analysis was performed on the 32 Karat™ 10.1 software. Sample preparation and instrument setup were carried out according to the manufacturer's application guide, RUO-IDV-05-6934-A (Sciex, Framingham, U.S.A.) for SDS-MW analysis kit, (part number 390953) (Sciex, Framingham, U.S.A.). A total 10 μL of sample was mixed with 85 μL of SDS-MW sample buffer and 5 μL of β-mercaptoethanol (BME) to denature by incubating at 95 °C for 5 min. The SDS-MW size standard was prepared similarly with the modification of adding 2 μL of a 10 kDa internal standard in the mix. Denatured samples were centrifuged at 15,000 ×*g* and transferred to micro-PCR tubes for CGE loading. Samples and standards were injected sequentially into cathode side of the SDS-MW gel buffer filled capillary for separation at 15 kV for 35 min at 25 °C. The UV absorbance was monitored at 214 nm during separation of the sample. For each run, capillary was rinsed first followed by 3 min wash with 0.1 N NaOH, 1 min with 0.1 N HCl, 1 min with water, then conditioned with SDS-MW gel buffer for 10 min with 70 psi pressure at the cathodic side. The capillary tips were dipped into water after loading with the capillary with SDS-MW gel buffer and sample injection to avoid contamination. Collected data were integrated and the molecular weights were analyzed according to the standards calibration.

### 2.4 Protein extraction and sample preparation for mass spectrometry

A total of 10 mg of sample was mixed with lysis buffer in 1:50 (w:v) ratio containing 50 mM TEAB and 5 % SDS of pH 8. Samples were mixed vigorously and incubated for 15 min at room temperature. The protein concentration was determined using RC DC™ Protein Assay by following manufacture's protocol. For protein digestion, 300 μg of total protein was mixed with trypsin enzyme in 50:1 (w:w) ratio for digestion utilizing S-Trap™ mini columns according to the manufacturer's recommendations. After digestion, total peptides were quantified by Pierce™ Quantitative Colorimetric Peptide Assay. Aliquots of 100 μg total peptide from each sample was freeze dried and reconstituted with 100 μL of 2 % acetonitrile (ACN) and 0.1 % formic acid (FA) (Fang et al., 2024).

### 2.5 Shotgun mass spectrometry (LC-MS/MS)

Two technical repeats of peptide digests from two biological replicate samples were injected into Q Exactive™ orbitrap mass spectrometry (MS) (Thermo Scientific™, Mississauga, ON, Canada) coupled with Vanquish UHPLC system (Thermo Scientific™, Mississauga, ON, Canada) set in Data Dependent Acquisition (DDA) mode. A total 50 μg of sample was injected into the Vanquish UHPLC system connected to C18 reverse phase Acclaim PepMap (1x150mm, 2 μm) column. The gradient was generated by mobile phase A (0.1 % formic acid in water) and mobile phase B (80 % acetonitrile with 0.1 % formic acid) as follows: 4–10 % of solvent B for 4 min, followed by 10–40 % of solvent B for next 79 min, 40–65 % of solvent B for 5 min, 65–100 % solvent B in 2 min, hold at 100 % of solvent B for 10 min and back to 4 % solvent B in 1 min at flow rate of 50 μL/min. Elution from the column was injected directly into the Q Exactive™ orbitrap MS under positive ion mode. At full MS, precursor ions were acquired across the scan range of 350–1800 *m*/*z* at high-resolution mode (resolution >70,000), AGC target 3e6 and accumulation time of 200 ms. For product ion selection and detection under dd-MS^2^ (TopN), top 12 most abundant precursors in each duty cycle were selected. The MS/MS fragmentation was performed in high sensitivity mode (resolution >17,500), AGC target-2e5, accumulation time 220 ms, loop count-12, isolation window-110 m/z and 20 s of dynamic exclusion.

### 2.6 Protein identification

The raw MS data files were analyzed using PEAKS Studio 10.6 build 20,201,221 (Bioinformatics solutions Inc.) software. Protein databases of bacteria, fungi, chickpea (*Cicer arietinum*), lentil (*Lens culinaris*) and faba bean (*Vicia faba*) were downloaded from uniprot server (https://www.uniprot.org/). Common contaminant proteins found in proteomics experiments were downloaded from the common repository of adventitious proteins (cRAP) database from “The Global Proteome Machine” (https://www.thegpm.org/crap/). For protein identification, following search parameters were used: parent mass error tolerance: 10.0 ppm, fragment mass error tolerance: 0.5 Da, precursor mass search type: monoisotopic, enzyme: trypsin, max missed cleavages: 3, digest mode: semi-specific, and false discovery rate (FDR): 1 %. For identification of protein groups from both substrate and microbial proteins, at least 1 unique peptide and 1 % FDR cut-off were used during initial database search. To identify and separate significant peaks from background noise signal, a peak intensity cut-off threshold value of 2E5 was used. The percentage distribution of protein from each group of organisms was calculated by using the assigned peak area of each protein. Since the same peptides were conserved among orthologous proteins from multiple species, a cut-off of at least 2 unique peptides per protein was applied to remove redundant identification for the calculation of relative protein abundances. A statistical measure -10lgP (minus 10 times the base 10 logarithm of the *P-value*) was used to assess the confidence of protein identifications. A cut-off for -10lgP score for peptide was set for ≥15, where 15 corresponds to a *P-value* of <0.032. Protein abundance was calculated as average of peak area ± standard deviation. To find the significant difference (*P-value* < 0.05), a one-way ANOVA with Tukey-Kramer post hoc test was performed (MS Excel, Microsoft, USA).

## 3 Results

### 3.1 Changes in the size distribution profiles of substrate proteins due to spontaneous fermentation

**Fig. 1:**
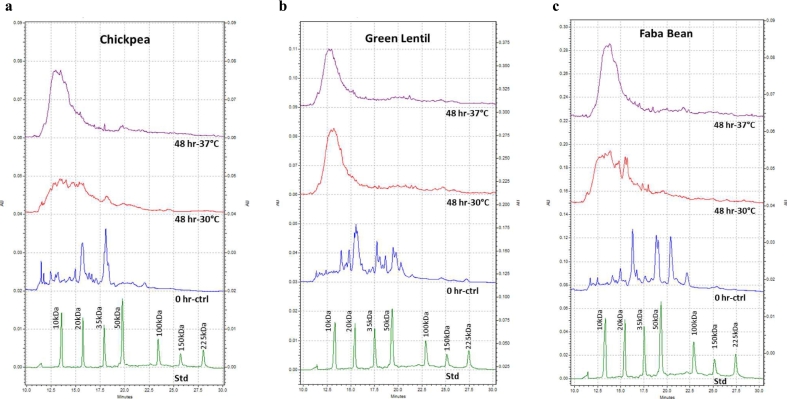
Analysis of protein hydrolysis by capillary gel electrophoresis (CGE) of control and spontaneous fermentation samples of a - chickpea protein isolate, b - green lentil protein isolate, and c - faba bean protein isolate. Each substrate was prepared and allowed to spontaneously ferment at two different temperatures (30 °C and 37 °C) for 48 h. The lower dark green trace indicates the migration of a standard calibration mix (Std) analyzed under the same conditions as the samples. X-axis denotes electrophoretic separation time in minutes and Y-axis denotes absorbance unit at 214 nm (AU). (For interpretation of the references to colour in this figure legend, the reader is referred to the web version of this article.)

The CGE chromatogram of the unfermented 0 h-ctrl isolates showed the presence of multiple protein peaks across a wide molecular weight range in all three protein isolate substrates. The unfermented chickpea protein isolate showed the presence of mostly smaller proteins that were below the 50 kDa size range, whereas, both the green lentil and faba bean protein isolates had prominent protein peaks in between the 50 to 100 kDa range (Fig. 1). It should be noted that all the samples in this analysis were reduced and denatured prior to analysis through the addition of β-mercaptoethanol and sodium dodecyl sulfate followed by incubation at 95 °C. This would ensure that the inter-molecular complexes between proteins were disrupted and the peaks observed in these chromatograms represent the monomeric molecular sizes of different classes of proteins present in the isolates. All the spontaneously fermented samples, showed a reduction in the peaks at higher molecular weight ranges and a corresponding increase in signal intensity at the lower molecular weight ranges. This left shift in protein peaks associated with their reduction in molecular weight distribution, is indicative of their proteolysis. This change in molecular size distribution suggests that the proteins have been broken down into smaller peptides or amino acids as a result of production of hydrolytic enzymes by the microbes growing on the substrate. The chickpea and faba bean protein isolates showed some level of resistance to proteolysis at the 30 °C incubation temperature compared to 37 °C, in which they had undergone more hydrolysis (Fig. 1a and c). However, the lentil protein isolate was susceptible to proteolytic processing even at the lower incubation temperature (Fig. 1b). Since these 48 h time-point samples were derived from uninoculated substrates, it can be hypothesized that these substrates might have been successfully colonized by endogenous microbes leading to the hydrolytic processing of the substrate proteins by the enzymes secreted by these microbes. To identify the changes occurring to proteins during spontaneous fermentation, further proteomics studies using mass spectrometry-based techniques were carried out.

### 3.2 Number of proteins identified from the substrate matrix before and after fermentation

**Fig. 2:**
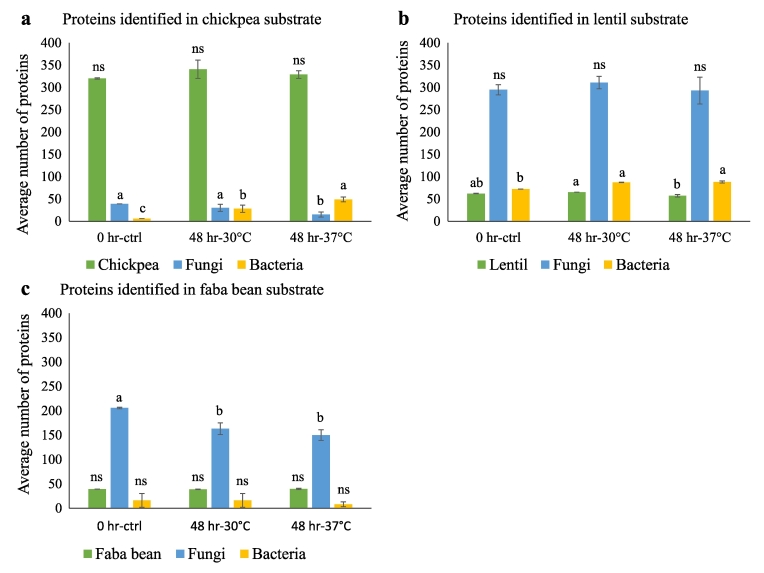
Number of unique protein groups identified from control and spontaneously fermented samples collected from a - chickpea isolate substrate, b - green lentil isolate substrate, and c - faba bean isolate substrate. Each bar represents the average number of protein groups identified from four replicates. X-axis denotes sample time points 0 h-control, 48 h-30 °C and 48 h-37 °C and Y-axis denotes the average number of proteins from substrate (green), fungi (blue) and bacteria (yellow) species from each sample. Lowercase letters above the bars indicate significant differences (*p value* < 0.05). ns- not significant. (For interpretation of the references to colour in this figure legend, the reader is referred to the web version of this article.)

To identify proteins present in the substrate matrices before and after fermentation, LC-MS/MS analysis was conducted on the control and spontaneously fermented samples from chickpea, lentil, and faba bean protein isolates incubated at two different temperatures, 30 °C and 37 °C. The analysis revealed the presence of hundreds of unique protein groups specific to the chickpea, lentil, and faba bean substrates, as well as the occurrence of unique protein groups from diverse bacterial and fungal species (Fig. 2 and Supplementary file).

In the 0 h-ctrl samples from chickpea, the majority of proteins identified were from the chickpea substrate (320 protein groups), followed by fungal proteins (39 protein groups) and very low numbers of bacterial proteins (6 protein groups) (Fig. 2a). However, after 48 h of spontaneous fermentation at 30 °C, the number of proteins from bacteria and fungi became almost similar at 28 and 30 protein groups, respectively, while at fermentation at 37 °C, the number of bacterial proteins significantly increased further (49 protein groups) with an associated significant reduction in the numbers of fungal proteins to 15 protein groups (Fig. 2a). This is probably because higher temperatures might favour bacterial growth, enhancing their metabolism and proliferation. This could allow them to outcompete fungi by consuming more nutrients, leading to increased bacterial protein production, while simultaneously reducing fungal growth (Emkani et al., 2022; Sáez et al., 2018). Such shifts in microbial populations have been shown to significantly impact the functional properties and flavor profiles (W. Li & Wang, 2021; Mefleh et al., 2022). In contrast, fewer proteins were identified from faba bean and lentil substrates, probably due to the limited number of protein sequence information available in their corresponding UniPortKB proteome databases compared to chickpea (total number of sequences available in UniProt database: faba bean – 9789, lentil – 900, and chickpea – 44,456). The 0 h-ctrl samples from lentil protein isolates had a much higher number of fungal (294 protein groups), as well as bacterial proteins (72 protein groups) compared to lentil proteins (62 protein groups) (Fig. 2b). While the number of fungal proteins did not show any significant changes at both incubation temperatures, the number of bacterial proteins increased significantly at both fermentation temperatures compared to the 0 h-ctrl samples (Fig. 2b). Similar to the lentil control, the 0 h-ctrl faba bean sample showed a high number of fungal proteins (206 protein groups) compared to the substrate proteins (39 protein groups) (Fig. 2c). However, in both the 48 h fermented faba bean samples the number of fungal proteins were significantly lower compared to the 0 h-ctrl (Fig. 2c), whereas, the number of bacterial proteins did not change significantly. A general trend observed in these results show a slight reduction in the number of fungal proteins at the higher 37 °C incubation temperature. Also, the number of bacterial proteins showed significant increases in the chickpea and lentil fermented samples compared to the controls. Finally, the faba bean samples did not follow this trend of increase in bacterial proteins after fermentation and this correlates with the higher resistance to proteolysis observed in the CGE size distribution profiles (Fig. 1c).

### 3.3 Relative abundance of proteins in the substrate matrix

**Fig. 3:**
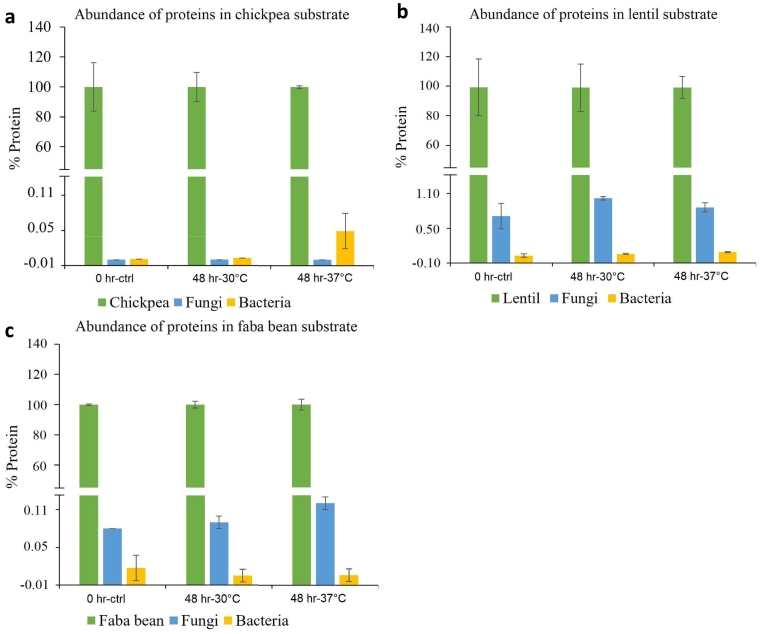
Abundance and relative distribution of proteins from substrate, fungal, and bacterial sources from control and spontaneously fermented samples collected from a - chickpea isolate substrate, b - green lentil isolate substrate, and c - faba bean isolate substrate. X-axis denotes sample time points 0 h-control, 48 h-30 °C and 48 h-37 °C and Y-axis denotes the percentage protein distribution (% protein) from substrate (green), fungi (blue) and bacteria (yellow) species from each sample. (For interpretation of the references to colour in this figure legend, the reader is referred to the web version of this article.)

Since the number of proteins identified may not indicate the abundance of proteins from different organisms, the peak area information from mass spectrometry data was analyzed to calculate the abundance percentage of proteins from pulse protein isolate substrate, fungal and bacterial origins (Fig. 3). The unfermented chickpea protein isolate control showed the lowest level of microbial contamination, followed by faba bean and lentil controls. The relative abundance of bacterial proteins in the substrate increased in the spontaneously fermented chickpea samples, with the 37 °C incubation temperature having a higher abundance of bacterial proteins (0.05 %) compared to the control (0 %) and 30 °C samples (0.003 %) (Fig. 3a). This suggests a colonization of the chickpea substrate primarily by bacterial species. The unfermented lentil protein isolate control contained a high amount of fungal protein, at 0.71 % of the total protein compared to the other two substrates (Fig. 3b). The relative amounts of fungal proteins increased in the spontaneously fermented lentil samples at 30 °C and 37 °C to 1.03 % and 0.86 %, respectively, suggesting successful colonization of the lentil substrate by fungal species. Additionally, the abundance of the bacterial proteins increased to 0.049 % in the fermented lentil sample at 37 °C compared to the unfermented lentil control. The unfermented faba bean substrate showed a relative bacterial protein abundance of 0.017 % and fungal protein abundance of 0.08 % (Fig. 3c). A trend of increase in fungal protein abundance and a decrease in bacterial protein abundance was observed with fermentation of the faba bean substrate. It is important to note that while the total number of substrate proteins identified in the lentil and faba bean samples were lower than the total number of microbial proteins (Fig. 2b and c), the relative abundance calculations clearly show that almost 99 % of all these samples were composed of substrate proteins (Fig. 3b and c), with a relatively low abundance (less than 1 %) of microbial proteins even after 48 h of fermentation. Further, due to this extremely low abundance of microbial proteins in comparison to the substrate proteins, as well as the biological variability in the sample replicates, the differences observed between the unfermented and fermented samples were not statistically significant and these relative abundance numbers should only be used for understanding the general trends of changes in microbial protein abundance.

### 3.4 High abundance proteins in substrate matrix

In terms of the individual proteins that were identified, not surprisingly, the most abundant proteins present in all the samples from all three substrates were the globulin class of seed storage proteins, such as vicilin and legumin (Table 2, Table 3, Table 4 and Supplementary file). This correlates well with the relative abundance calculations that showed that approximately 99 % or more of the control as well as fermented samples belonged to the substrate proteins (Fig. 3). Among the non-substrate microbial proteins identified from the chickpea substrate, fungal proteins associated with the ribosomal subunits (40S ribosomal protein S23 from *Candida tenuis*) and cellular structure (ADP-ribosylation factor from *Cryptococcus neoformans* and *Malassezia sympodialis*) were the most abundant at the 0 h time-point samples (Table 2). The 48 h chickpea samples that were spontaneously fermented at 30 °C still had an abundance of fungal ADP-ribosylation factor proteins from *Pichia kudriavzevii*. However, as observed in the number of proteins (Fig. 2a) as well as their relative abundance (Fig. 3a), the bacterial protein, Elongation factor Tu, from several *Bacillus* sp. was the most abundant non-substrate protein identified in the chickpea substrate fermented for 48 h at 37 °C (Table 2). The most abundant non-substrate protein found in the 0 h lentil sample was the fungal protein Histone H2A from *Phaffia rhodozyma* (Table 3). The same proteins were also the most abundant ones identified from the 48 h spontaneously fermented lentil samples incubated at both 30 °C and 37 °C. This is in good agreement with the observation that both the number and abundance of fungal proteins remained higher than the bacterial proteins in both temperatures in the lentil substrate (Fig. 2b and 3b). The non-substrate proteins identified from faba bean substrate samples collected at all time-points and temperatures were Histone H4 from fungal species such as, *Cryptococcus neoformans* and *Trichosporon asahii* (Table 4), again in good correlation to the observation that the number and relative abundance of fungal proteins were higher than the bacterial proteins in all the faba bean samples (Fig. 2c and 3c). Although microbial species have been indicated based on the proteins that have been identified through this proteomics-based approach, it should be noted that there are limitations for precise identification and taxonomic profiling of microorganisms, as proteins sequence might be well conserved across different microbial species, making it challenging to accurately identify the correct microorganism based solely on protein profiles (Chandramouli & Qian, 2009). However, the approach used here not only provides the capacity to identify proteins from diverse microbial species, but also obtain information about the substrate proteins and their relative abundance simultaneously, which will not be possible by other genomics methodologies. Further, as these samples have undergone heat treatments post-fermentation, the quality of the nucleotide sequence available from these substrates would be sub-optimal for such approaches. Therefore, integration of data from the current study with other omics technologies such as metagenomics, transcriptomics, and metabolomics can provide a complete picture of the changes in proteome during spontaneous fermentation as well as the identity of the specific organisms causing those changes (Aguiar-Pulido et al., 2016).

**Table 2:** List of proteins identified from unfermented and spontaneously fermented chickpea samples.

Top 3 abundant chickpea proteins from all samples											
Accession	Description	Origin	-10lgP	Area VSC1	Area VSC2	Area VSC3	Area VSC4	Area VSC5	Area VSF6	#Peptides	#Unique
A0A1S2XQR4	vicilin-like	Chickpea	469.5	1.22E+09	2.43E+09	2.17E+09	1.95E+09	1.22E+09	9.53E+08	251	41
A0A1S2XSB9	legumin A-like	Chickpea	459.84	1.84E+09	2.95E+09	3.04E+09	2.49E+09	1.66E+09	1.74E+09	216	136
A0A3Q7XNW1	legumin-like	Chickpea	436.65	1.46E+09	1.92E+09	2.18E+09	1.71E+09	8.09E+08	1.02E+09	209	41


Top 3 abundant microbial proteins from 0-h control samples							
Accession	Description	Origin	-10lgP	Area VSC1	Area VSC2	#Peptides	#Unique
G3B4T6	40S ribosomal protein S23	Fungi	43.36	1.07E+06	1.33E+06	1	1
A0A5Q7YLL1, P0CM17, A0A061AUV5, M5E5Q4, A0A0F7ST88, A0A099NYT5, W3VQ51, M7XGC1	ADP-ribosylation factor 1	Fungi	83.64	3.48E+05	4.64E+05	4	4
F5HAS0, A0A5C3FI26, A0A0K3CQA4, A0A0J0XN23, V5ER47, A0A8H3YHF1, A0A0P9EI62, A0A0E9NM83	Vacuolar proton pump subunit B	Fungi	48.34	1.10E+05	1.49E+05	4	4


Top 3 abundant microbial proteins from 48-hour 30°C fermentation samples							
Accession	Description	Origin	-10lgP	Area VSC3	Area VSC4	#Peptides	#Unique
A0A5Q7YLL1, B9WE85, A0A8H7ZI61, C5MAF4, A0A5C3FF32, W3VQ51, A0A099NYT5, A0A060T7Q4, A0A061AUV5, M5E5Q4, A0A8H3TPZ7, A0A0F7ST88, M7XGC1	ADP-ribosylation factor	Fungi	83.64	3.66E+05	1.66E+05	4	4
F5HCP2, F5HFP4, Q5K7W2, A0A8H3YHX5, A0A5C3FH98, M9M4T4	ADP/ATP translocase	Fungi	73.54	3.51E+05	1.02E+05	4	4
A0A0K3CQA4, A0A511KLX7, A0A061BL90, A0A2S9ZX33, M7X730, A0A0P9EI62, A0A5C3FI26, R9P4L7, F5HAS0, Q5KBW0, A0A0E9NM83, V5ER47, A0A0J0XN23, A0A8H3YHF1	V-type proton ATPase subunit B	Fungi	48.34	2.30E+05	3.81E+04	3	3


Top 3 abundant microbial proteins from 48-hour 37°C fermentation samples							
Accession	Description	Origin	-10lgP	Area VSC5	Area VSC6	#Peptides	#Unique
Q81VT2, C3P9Q3, C3LJ80, C1ET37, B7HQU2, B7JKB7, Q73F98, Q814C4, B7HJ46, B9IZJ2, Q63H92, A0R8H8, Q6HPR0, B7IT17, A9VP75	Elongation factor Tu	Bacteria	111.92	5.42E+05	3.09E+06	7	7
Q81VE1, C3PAV1, C3L507, C1EUB1, B7HS05, B7JM60, Q73ER9, B9J1H2, Q63GV7, A0R8W4, Q6HPC7	Chaperonin GroEL	Bacteria	125.21	5.93E+04	2.03E+06	14	13
A0A5Q7YLL1, B9WE85, A0A8H7ZI61, C5MAF4, A0A5C3FF32, W3VQ51, A0A099NYT5, A0A060T7Q4, A0A061AUV5, M5E5Q4, A0A8H3TPZ7, A0A0F7ST88, M7XGC1	ADP-ribosylation factor	Fungi	83.64	5.19E+05	1.73E+06	4	4

**Table 3:** List of proteins identified from unfermented and spontaneously fermented lentil samples.

Top 3 abundant lentil proteins from all samples											
Accession	Description	Origin	-10lgP	Area VSL1	Area VSL2	Area VSL3	Area VSL4	Area VSL5	Area VSL6	#Peptides	#Unique
Q84UI1	Allergen Len c 1.0101 (Fragment)	Lentil	422.99	8.68E+08	1.52E+09	4.98E+08	7.04E+08	4.47E+08	4.18E+08	217	60
Q84UI0	Allergen Len c 1.0102 (Fragment)	Lentil	411.32	2.41E+08	4.19E+08	2.17E+08	2.65E+08	2.03E+08	2.23E+08	185	38
P13918	Vicilin	Pea	383.67	8.30E+08	9.93E+08	6.11E+08	8.82E+08	5.72E+08	6.95E+08	164	88


Top 3 abundant microbial proteins from 0-h control samples							
Accession	Description	Origin	-10lgP	Area VSL1	Area VSL2	#Peptides	#Unique
A0A0F7SUV9, A0A0F7SM42, A0A0F7SGU4, A0A8H3TP00, V5EWU2, M5E9U5, P02264, M9LZI8, C0HJQ3, P0CN99, P0CN98, A0A0J0XJD6, A0A0J0XVS1, A0A0J0XKL2, A0A5C3FH76, R9P5C6, A0A061B9S1, A0A0K3CFH9, G0SZA2, A0A194S7P0, P84056, J5TNA7, J6F0U6, K1VQ33, K1VQJ6, Q4PEF9, Q4PHE4, Q6C341, A0A8J5BU84, A0A8J5BT80	Histone H2A	Fungi	46.6	9.69E+06	2.73E+07	2	2
A0A835YJ82	Histone H4	Fungi	156.61	7.29E+06	6.73E+06	7	7
G3AX77, G3B0Y9, B6K1Z1	Ubiquitin	Fungi	111.13	3.87E+06	3.24E+06	5	5


Top 3 abundant microbial proteins from 48-h 30 °C fermentation samples							
Accession	Description	Origin	-10lgP	Area VSL3	Area VSL4	#Peptides	#Unique
A0A0F7SUV9, A0A0F7SM42, A0A0F7SGU4, A0A8H3TP00, V5EWU2, M5E9U5, P02264, M9LZI8, C0HJQ3, P0CN99, P0CN98, A0A0J0XJD6, A0A0J0XVS1, A0A0J0XKL2, A0A5C3FH76, R9P5C6, A0A061B9S1, A0A0K3CFH9, G0SZA2, A0A194S7P0, P84056, J5TNA7, J6F0U6, K1VQ33, K1VQJ6, Q4PEF9, Q4PHE4, Q6C341, A0A8J5BU84, A0A8J5BT80	Histone H2A	Fungi	46.6	2.01E+07	1.67E+07	2	2
A0A835YJ82	Histone H4	Fungi	156.61	8.60E+06	1.19E+07	7	7
G3AX77, G3B0Y9, B6K1Z1	Ubiquitin	Fungi	111.13	4.93E+06	4.82E+06	5	5


Top 3 abundant microbial proteins from 48-h 37 °C fermentation samples							
Accession	Description	Origin	-10lgP	Area VSL5	Area VSL6	#Peptides	#Unique
A0A0F7SUV9, A0A0F7SM42, A0A0F7SGU4, A0A8H3TP00, V5EWU2, M5E9U5, P02264, M9LZI8, C0HJQ3, P0CN99, P0CN98, A0A0J0XJD6, A0A0J0XVS1, A0A0J0XKL2, A0A5C3FH76, R9P5C6, A0A061B9S1, A0A0K3CFH9, G0SZA2, A0A194S7P0, P84056, J5TNA7, J6F0U6, K1VQ33, K1VQJ6, Q4PEF9, Q4PHE4, Q6C341, A0A8J5BU84, A0A8J5BT80	Histone H2A	Fungi	46.6	9.27E+06	1.22E+07	2	2
A0A835YJ82	Histone H4	Fungi	156.61	8.56E+06	1.04E+07	7	7
G3AX77, G3B0Y9, B6K1Z1	Ubiquitin	Fungi	111.13	3.42E+06	3.98E+06	5	5

**Table 4:** List of proteins identified from unfermented and spontaneously fermented faba bean samples.

Top 3 abundant faba bean proteins from all samples											
Accession	Description	Origin	-10lgP	Area VSF1	Area VSF2	Area VSF3	Area VSF4	Area VSF5	Area VSF6	#Peptides	#Unique
I0B569	Vicilin	Faba bean	375.94	4.43E+09	4.00E+09	4.07E+09	4.53E+09	3.76E+09	3.21E+09	204	171
P05190	Legumin type B	Faba bean	375.35	1.47E+09	1.74E+09	1.82E+09	1.69E+09	1.96E+09	1.92E+09	193	67
Q99304	Legumin A2 primary translation product	Faba bean	344.21	1.62E+09	1.82E+09	1.72E+09	1.68E+09	1.28E+09	1.37E+09	156	72


Top 3 abundant microbial proteins from 0-h control samples							
Accession	Description	Origin	-10lgP	Area VSF1	Area VSF2	#Peptides	#Unique
A0A835YJ82	Histone H4	Fungi	128.83	3.87E+06	4.34E+06	9	5
P62792, A0A0J1BCP9, A0A0F7SLM7, Q5K8H5, J6EWV4, F5HA66, K1VI65	Histone H4	Fungi	95.39	9.07E+06	8.50E+06	5	1
R7YN28, H6C6P4, A0A0N1HXH2	Molecular chaperone HtpG	Fungi	77.54	1.22E+06	7.37E+05	3	3


Top 3 abundant microbial proteins from 48-h 30 °C fermentation samples							
Accession	Description	Origin	-10lgP	Area VSF3	Area VSF4	#Peptides	#Unique
A0A835YJ82	Histone H4	Fungi	128.83	4.45E+06	3.54E+06	9	5
P62792, A0A0J1BCP9, A0A0F7SLM7, Q5K8H5, J6EWV4, F5HA66, K1VI65	Histone H4	Fungi	95.39	9.32E+06	1.20E+07	5	1
R7YN28, H6C6P4, A0A0N1HXH2	Molecular chaperone HtpG	Fungi	77.54	6.65E+05	9.20E+05	3	3


Top 3 abundant microbial proteins from 48-h 37 °C fermentation samples							
Accession	Description	Origin	-10lgP	Area VSF5	Area VSF6	#Peptides	#Unique
A0A835YJ82	Histone H4	Fungi	128.83	6.10E+06	5.67E+06	9	5
P62792, A0A0J1BCP9, A0A0F7SLM7, Q5K8H5, J6EWV4, F5HA66, K1VI65	Histone H4	Fungi	95.39	1.49E+07	1.16E+07	5	1
R7YN28, H6C6P4, A0A0N1HXH2	Molecular chaperone HtpG	Fungi	77.54	1.12E+06	7.83E+05	3	3

## 4 Discussion

Many bacteria, filamentous fungi and yeast species naturally ferment foods and beverages. Lactic acid bacteria (LAB) such as *Lactobacillus*, *Lactococcus*, *Leuconostoc*, *Streptococcus*, *Pediococcus* are the most common genera of gram-positive bacteria identified from various fermented foods and beverages (Axelsson et al., 2012; Holzapfel & Wood, 2014; Salminen & Von Wright, 2004). Genera of fungus like *Aspergillus*, *Brettanomyces*, *Candida*, *Cryptococcus*, *Neurospora*, *Pichia*, *Rhodotorula*, *Rhodosporidium*, *Saccharomyces* and *Zygosaccharomyces* have been identified in many fermented foods and beverages (Tamang et al., 2016). Traditional foods that are produced by spontaneous fermentation due to the presence of many of the above-mentioned microbes have a long history of safe consumption by humans. Characterizing the native species of microbes involved in spontaneous fermentation and identifying the proteins added to the fermented substrates by these microbes is essential for ensuring food safety for new applications being developed.

### 4.1 Changes in nutritional and functional properties of substrate associated with modifications in protein size distribution during spontaneous fermentation

In a recent study published by this team, three pulse protein isolate substrates belonging to chickpea, green lentil and faba bean were inoculated with known strains of fungal and bacterial species and the effects of such inoculations were investigated when the substrates were incubated at 30 °C and 37 °C (Stone et al., 2024). The choice of incubation temperatures for the SSF is primarily based on previously optimized growth conditions for the fungal (*Aspergillus* sp.) and bacterial (*Lactobacillus* sp.) species that were used for the inoculated fermentation study (Batbayar et al., 2023; Khorsandi, Shi et al., 2024; Kumitch et al., 2020). Interestingly, it was observed that the uninoculated pulse protein substrates showed an increased degree of hydrolysis when then they were incubated at 30 °C and 37 °C for 48 h compared to the 0 h control samples (Stone et al., 2024). This increase in degree of hydrolysis of proteins in uninoculated substrates indicated the occurrence of spontaneous fermentation, prompting the current investigation into identifying the proteins produced and studying the proteome-level changes. The CGE analysis performed in the current study revealed drastic reduction in molecular size distribution of spontaneously fermented substrates, correlating very well with the increased degree of hydrolysis observed in the previous study. Similar reduction in protein size distribution through hydrolysis has been previously reported in studies where pulse protein substrates were intentionally inoculated with bacterial or fungal strains in SSF as well as submerged fermentation processing (Khorsandi, Stone et al., 2024; D. Shi et al., 2024; Y. Shi et al., 2021). The previous study also revealed that the spontaneously fermented substrates showed a significant increase in protein solubility and total phenolic content, but a significant decrease in the in vitro protein digestibility when compared to the unfermented 0 h controls. The water and oil holding capacities of these spontaneously fermented substrates did not show changes compared to the controls. The digestibility of proteins could be influenced by various factors, such as the nature of protease enzymes secreted by the microbes, composition of substrate proteins, resistance of substrate proteins to hydrolysis, presence of anti-nutritional factors, and levels of starch and lipids (Emkani et al., 2022; Senanayake et al., 2023). However, a significant increase in the total phenolic content was recorded in all the fermented samples and it was hypothesized that this might have reduced their in vitro digestibility due to non-specific interactions and inhibition of the digestive enzymes used (Stone et al., 2024).

In a previous study investigating the effects of solid-state fermentation of pea protein isolate inoculated with the fungus *Aspergillus oryzae*, a similar mass spectrometry-based proteomics approach shed insights into the number of proteins originating from the substrate and the inoculated microbe at increasing time points of fermentation, the relative abundance of substrate proteins and *A. oryzae* proteins and the molecular networks that were involved in the growth of the microbe and modification of the substrate (Das et al., 2023). Interestingly in that study, it was observed that the relative abundance of the proteins from the inoculate *A. oryzae* increased from 0.79 % at the time of inoculation to 6.8 % at 36 h post inoculation. In comparison to those results, the current study revealed that the relative abundance of all the bacterial and fungal proteins combined together reached a level of approximately only 1 % of total proteins in the spontaneously fermented lentil substrates. The abundance of the microbial proteins was even lower in the fermented chickpea and faba bean substrates. It is interesting to note that although the total abundance of microbial proteins was relatively low in the spontaneously fermented samples compared to the increased abundance noted in a similar analysis of an inoculated fermentation of a pea protein isolate substrate, the native microbes were able to modify the size profiles, functionality, digestibility and flavor of the substrates, as hydrolytic enzymes produced by these microbes might successfully exert their activity on substrate proteins at the temperature of incubation and in the time period provided.

### 4.2 Nature of proteins and microbes found in different substrates

Interestingly, all the unfermented 0 h control substrates showed the presence of multiple bacterial and fungal proteins belonging to diverse species. This finding is in good agreement with a recent study showing the presence of viable bacterial species that were isolated and cultured from a diverse collection of plant-based ingredients (Kyrylenko et al., 2023). That study had collected and analyzed 88 commercial ingredients, such as flours, concentrates and isolates from pea, faba bean, chickpea and several other non-legume plant-based ingredients. However, that study did not explore the possibility of these microbes growing on the substrates and modifying them if the right environment is provided to them. Here we report the identification of a diversity of proteins produced by these endogenous microbes as they grow and spontaneously ferment the substrates.

In the chickpea substrate, the most abundant fungal protein identified after spontaneous fermentation at both 30 °C and 37 °C was ADP-ribosylation factor from the fungus *Pichia kudriavzevii*. Generally, *Pichia* species are ubiquitous and involved in beverage and food fermentations (Chu et al., 2023). In the spontaneous fermentation of chickpea substrate at 37 °C, both the number and abundance of bacterial proteins overtook the fungal ones, supporting the hypothesis that the higher fermentation temperatures might favour growth of bacteria over fungal species. Further, there could be competition between various microbial species trying to compete for nutritional resources from the substrate. Many traditional spontaneous fermentative processing has relied on the use of LAB species that can acidify the substrate through the production of lactic acid from sugars. This acidification is known to cause the inhibition of many pathogenic microbial species and act as a traditional food preservation tool (Sionek et al., 2023; Thierry et al., 2023). The most abundant bacterial proteins identified from the chickpea substrate incubated at 37 °C were from several *Bacillus* species. *Bacillus* species are associated with fermentative processing, as well as spoilage of foods and concerns of food safety (André et al., 2017; Stenfors Arnesen et al., 2008). Certain *Bacillus* species such as specific *B. subtilis* strains are used in the production of natto, a fermented food from East Asia. They are also utilized in the production of proteases, antimicrobial substances, for the creation of distinctive flavors and aromas in fermented foods, enhancement of nutrients, and preservation of food (Z. Li et al., 2023; Sivamaruthi et al., 2022).

In all the samples collected from unfermented and fermented lentil substrates, proteins from fungal species dominated both in numbers and abundance. In contrast to chickpeas, lentil controls began with a significantly higher abundance and number of proteins from fungal species compared to bacterial species. This variation in the microbial protein composition of the lentil compared to chickpeas could be due to various factors, such as agricultural practices, growth and storage environment, post-harvest processing conditions, as well as the unique molecular compositions of the substrate that could selectively favour one group of organisms over the others. The most abundant protein from both the unfermented controls, as well as the samples from fermentation at 30 °C and 37 °C in the lentil substrate was Histone H2A from the fungi *Phaffia rhodozyma*. This is a carotenoid-producing yeast species that is attracting attention for the commercial production of the pigment astaxanthin that is utilized in aquafeed applications (Johnson, 2003).

Similar to lentil substrate, the abundance of fungal proteins was higher than bacterial proteins in unfermented faba bean samples. The most abundant protein from the unfermented as well as both the spontaneously fermented faba bean samples was Histone H4 from fungal species such as *Cryptococcus neoformans*, *Phaffia rhodozyma*, and *Trichosporon asahii*. Proteins from *Marinobacter nauticus* and *Thiobacillus denitrificans* were the most abundant bacterial proteins found in the unfermented as well as fermented faba bean samples. Interestingly, none of the proteins from *Bacillus* sp., which were abundant in the fermented chickpea samples, were identified from any of the faba bean samples, revealing the unique composition of endogenous microbial populations in different substrates and their influence on the progression of spontaneous fermentation in different paths.

Finally, most of the microbial proteins that were detected in all these spontaneously fermented substrates were common housekeeping proteins that would play a central role in growth, protein synthesis and cell division. This indicates that these organisms were probably still in their early to mid phase of growth curves after 48 h of incubation. In the earlier proteomics studies that was conducted on inoculated fermentation of pea protein isolate, it was observed that such housekeeping proteins dominated the proteome of the earlier fermentation time point of about 12 h post inoculation. However, at later time point of around 48 h, the proteome showed diversification into secondary metabolism pathways to support the metabolic reprogramming of late phase microbial growth (Das et al., 2023). This lag in growth rates reveals a key difference between the progression of microbial growth in inoculated fermentation versus uninoculated spontaneous fermentation. The inoculation of a large number (10^7^ spores per g of substrate) of freshly harvested microbial spores kickstarts the fermentation process and the inoculated species quickly establishes itself on the substrate and hence at 36 h post-inoculation the relative microbial protein abundance increased to 6.8 %. Whereas, probably due to the low number of viable endogenous microbes present in the uninoculated substrates, the spontaneous fermentation progresses at a slower rate and even after 48 h post-inoculation the relative microbial abundance is roughly about 1 %, that too in only one of the three substrates studied here.

## 5 Conclusion

This study, using a proteomics approach, has provided direct evidence of the presence of proteins produced by a diverse microbial population on three different plant-based protein substrates. Differences in the microbial communities were also observed between the different substrates. Further, the current work also characterized the changes in the numbers and abundance of these microbial proteins during spontaneous fermentation of the plant protein substrates. However, it should be noted that the relative abundance of proteins from the microbial populations present in all the samples tested was less than 1 % of the total protein. Even at this low relative abundance, the spontaneous fermentation was able to significantly impact the physicochemical and functional properties, as well as the flavor profiles of the protein ingredients. Although the approach used in this study will not be able to pinpoint the actual microbial species, except in cases where there are unique proteins or peptides that are specific to an organism, it has the capacity to directly identify the proteins present in the substrate. This could be beneficial in identifying certain proteins and peptides that could pose food safety concerns due to their allergenicity or toxicity. Hence, this should be used as a complementary approach to other techniques for characterizing microbial communities in foods.

## CRediT authorship contribution statement

**Prem Prakash Das:** Writing – original draft, Visualization, Validation, Software, Methodology, Investigation, Formal analysis, Data curation. **Xu Caishuang:** Methodology, Investigation, Formal analysis. **Lu Yuping:** Writing – review & editing, Methodology, Investigation. **Enyu Liu:** Resources, Methodology. **Zahra Jafarian:** Resources, Methodology. **Takuji Tanaka:** Supervision, Conceptualization. **Darren Korber:** Supervision, Conceptualization. **Michael Nickerson:** Writing – review & editing, Supervision, Funding acquisition, Conceptualization. **Nandhakishore Rajagopalan:** Writing – review & editing, Validation, Supervision, Project administration, Data curation, Conceptualization.

## Declaration of competing interest

The authors declare that they have no financial or personal relationships with other people or organizations that could inappropriately influence the work reported in this paper.

## Data Availability

Data will be made available on request.

## Appendix A Supplementary data

Supplementary material: Summary of proteins identified in samples from Chickpea (sheet 1), Lentil (sheet 2) and Faba bean (sheet 3) substrates. The data includes protein accession numbers, descriptions, species, confidence scores (-10lgP), quantitative areas for various samples, peptides, and unique peptides. Image 1
